# Relationship between the timing of physical therapy commencement and the duration of work disability: a retrospective cohort analysis of work-related low back pain claims

**DOI:** 10.1186/s12889-025-22574-x

**Published:** 2025-04-09

**Authors:** Tesfaye Hambisa Mekonnen, Luke R. Sheehan, Michael Di Donato, Alex Collie, Grant Russell

**Affiliations:** 1 Department of General Practice, School of Public Health and Preventive Medicine, Monash University, 553 St Kilda Road, Melbourne, 3004 Australia; 2 Department of Environmental and Occupational Health and Safety, Institute of Public Health, College of Medicine and Health Sciences, University of Gondar, P.O. Box. 196, Gondar, Ethiopia; 3 Healthy Working Lives Research Group, School of Public Health and Preventive Medicine, Monash University, 553 St Kilda Road, Melbourne, 3004 Australia

**Keywords:** Low back pain, Physical therapy, Return to work, Timing of care, Work disability, Workers’ compensation

## Abstract

**Background:**

Early physical therapy for workers reporting low back pain (LBP) may reduce disability and improve return to work. This study aimed to explore the relationship between the timing of physical therapy commencement and the duration of work disability after the onset of compensable LBP.

**Methods:**

We conducted a retrospective cohort analysis of workers with workers’ compensation claims for LBP in two Australian states. We investigated the association between the timing of physical therapy commencement and work disability duration using an accelerated failure time model. Median duration of work disability in paid calendar weeks was the principal outcome.

**Results:**

We examined 9160 accepted workers’ compensation claims for LBP. Patients who had not seen a physical therapist had the shortest duration of disability (median, 4.1 weeks). In those who had seen a physical therapist, the median duration of work disability was associated with the timing of commencement of physical therapy, from 8.0 weeks for care within 7 days of the injury to 34.7 weeks when care was commenced greater than 30 days after the onset of injury. Our adjusted model demonstrated that, compared to physical therapy within 7 days of injury onset, commencement of physical therapy between 8 and 14 days, 15 and 30 days, and greater than 30 days was associated with a 37.0% (Time ratios (TR) 1.37; 95% CI (1.23, 1.52)), 119% (TR 2.19; 95% CI (1.96, 2.44)) and 315% (TR 4.51; 95% CI (4.06, 5.02)) increased likelihood of longer disability duration, respectively.

**Conclusions:**

In workers with work-related LBP undertaking physical therapy, early commencement of physical therapy was associated with a significantly shorter duration of disability. Although we cannot establish causality, our findings highlight the potential benefits of initiatives that promote timely initiation of treatment in reducing extended work disability for injured workers undergoing physical therapy for LBP.

**Supplementary Information:**

The online version contains supplementary material available at 10.1186/s12889-025-22574-x.

## Background

Low back pain (LBP) remains a significant public health problem, affecting approximately 619 million people worldwide in 2020 [1]. In Australia, back problems represented the third most common cause of disease burden in 2023 [2]. LBP is the main contributor to the global burden of disability [3] and disproportionately impacts individuals during their work life [4]. LBP significantly contribute to time lost from work [3], and the resultant work disability presents considerable global health challenges [5]. Work-related disability places a profound economic strain on society through increased healthcare costs, disability insurance claims, and unemployment [6–9], and adversely affects individuals’ quality of life and social participation [8, 10].

Timely and effective treatment for LBP may be essential to reduce the risk of longer disability by promoting faster recovery and return to work [11, 12]. Practice guidelines often emphasise initial non-pharmacological interventions for LBP treatment [13]. Physical therapy (PT) may be considered as part of nonpharmacologic interventions for the management of acute LBP [14]. According to Lentz et al., timing of care (early versus late) is one of the core elements of value-based PT models that can influence outcomes for LBP [15]. However, the timing of initiating treatment is conceptually underemphasised in current practice guidelines [16]. A value-based model of PT highlights the importance of early intervention in the treatment process in improving patient outcomes [15]. This approach shifts the focus from the content of treatment to how care is delivered (whether through direct access or referral) and the timing of care, suggesting PT as the first option in the treatment sequence compared with alternative services.

Substantial evidence shows that early commencement of PT may effectively improve outcomes for individuals with LBP, including a reduction in work disability. A systematic review and meta-analysis conducted by McDevitt AW et al. demonstrated that early PT (i.e. PT initiated within 30 days after the date of the first visit) was associated with reduced short-term disability and pain (up to six weeks) in patients with acute LBP compared to non-PT individuals [16]. However, the study indicated minimal effect sizes. The investigators also found that early PT, compared to delayed PT, showed minor but non-significant tendencies of improved short-term outcomes (less than six months).

In Australia, individuals with work-related illnesses or injuries can access various supports for work disability via one of the eleven state-based workers’ compensation schemes. Each of these systems provides income support and funds considered reasonable and necessary for medical and rehabilitation services [17]. Injured workers have the right to choose their primary care provider for the treatment and management of their injuries and illnesses [18]. This includes allied healthcare professionals such as physiotherapists, osteopaths, and chiropractors [19–21]. Physiotherapists are common treatment providers for injured workers with LBP [22] and play a key role in facilitating their return to work [23].

The relationship between the timing of commencement of PT and return to work outcome after LBP has been little studied [24], particularly in a compensable environment. This gap in research exacerbates the lack of ideal timing for initiating PT in the current evidence base [25]. Gaining a comprehensive understanding of how the timing of PT affects patient outcomes following the onset of back pain could improve efforts to prevent work disability and minimise the related medical and nonmedical costs [26]. The current study evaluated the hypothesis that earlier PT commencement is associated with a shorter duration of work disability in a population of patients receiving workers’ compensation benefits following their injury.

## Research question

What is the relationship between the timing of PT commencement and work disability duration among workers with occupational-related LBP who have received PT services?

## Methods

### Study design and setting

In this study, we used administrative claims data from workers’ compensation authorities in two Australian states: South Australia and Western Australia [27]. As of the end of 2015, the total workforce in the two jurisdictions accounted for 18% of the labour force in Australia [28]. These two jurisdictions were selected as they contained all variables required to drive our outcome and exposure. The administrative data contains several demographic and occupational variables (e.g. sex, remoteness, time loss in weeks) at the claim level. It also contains service-level data such as jurisdiction, service date, injury date, and service descriptions. Procedures for cleaning and harmonisation of the dataset have been described elsewhere [27, 29, 30].

### Sample selection

We examined accepted workers’ compensation claims for LBP with injury dates between July 2011 and June 2015. Claimants were included if they were aged 15 to 80 years and off work for at least two weeks after injury. The Type of Occurrence Classification System (Supplementary file 1) [31] was employed to identify cases of LBP, as in earlier works [32, 33]. We restricted our analysis to services funded within two years of injury because, in some schemes in Australia, workers’ access to lost wage replacement ends after two years [34]. Only services involving direct patient interactions with clinicians, such as face-to-face consultations in clinics, and telehealth consultations were considered [27, 30, 35]. Physiotherapy, chiropractic and osteopathy are authorised to deliver services on behalf of both jurisdictions. We grouped these as representing physical therapy services.

### Study variables

#### Outcome

Our principal outcome was the median duration of work disability, measured in weeks. It is the number of weeks of wage replacements or income benefits paid for by the scheme. The sum of the total number of days paid for each claim is divided by 7 (standardisation) to calculate the median duration of work disability in paid calendar weeks [32].

#### Primary independent variable

The primary independent variable was the timing of PT commencement, defined as the number of days between the date of injury and the first visit to physical therapists. We categorised the timing of PT commencement into 0–7 days, 8–14 days, 15–30 days, > 30 days and no PT after injury, consistent with the literature [36].

#### Covariates

Several demographic and occupational covariates were available [29, 33]. Gender was classified as male versus female. Workers’ age at the time of injury was categorised into five groups:15–24 years, 25–34 years, 35–44 years, 45–55 years, and 55 + years, following previous publications [29, 35]. Socioeconomic status was categorised as most disadvantaged for scores in the lowest quintile and most advantaged for scores in the highest quintile according to the Index of Relative Socioeconomic Advantage and Disadvantage [37]. Following a previous study [29], the middle quintiles are combined into a single category. The Accessibility/Remoteness Index of Australia was used to group remoteness into major cities, regional, and remote [38] and the Australian and New Zealand Standard Classification of Occupations was used to classify occupations [39].

### Data analysis

Data for income replacement duration was skewed, so we used descriptive statistics to summarise data as the median (in weeks). An accelerated failure time (AFT) model was used to examine the relationship between PT timing and the duration of work disability [40]. We employed the AFT model because the assumption of proportional hazards was violated, and the proportional hazard assumptions are not required in AFT models [41]. The lognormal model was chosen based on the lower values (i.e. better model fit) of the Akaike Information Criterion and Bayesian Information Criteria [40].

The largest sample size within a category for all variables was used as a reference group. We used the time ratio (TR) to estimate the association between the timing of PT commencement and the duration of work disability. Findings are presented with corresponding CIs of 95%, where a TR > 1 indicates a longer duration of work disability. We plotted adjusted survival curves to compare the duration of work disability in weeks across the different PT initiation timing groups. We censored the work disability duration at 104 weeks. A p-value of < 0.05 was considered statistically significant. We employed multiple imputations by chained equation method (repeated the process 10 times) to replace missing data (accounted for less than 5% of the total dataset) for socioeconomic and remoteness variables. Stata version 17 was used for all analyses.

## Results

### Sample characteristics

A total of 9160 accepted workers’ compensation claims meeting eligibility criteria were included, with 36.6% (*n* = 3353) from South Australia and 63.4% (*n* = 5807) from Western Australia. Of the claimants, 87.0% (*n* = 7965) had at least one PT session following the injury date, whereas 13.0% (*n* = 1195) received no PT during the study period.

### Distribution of timing of PT commencement after injury onset

**Fig. 1:**
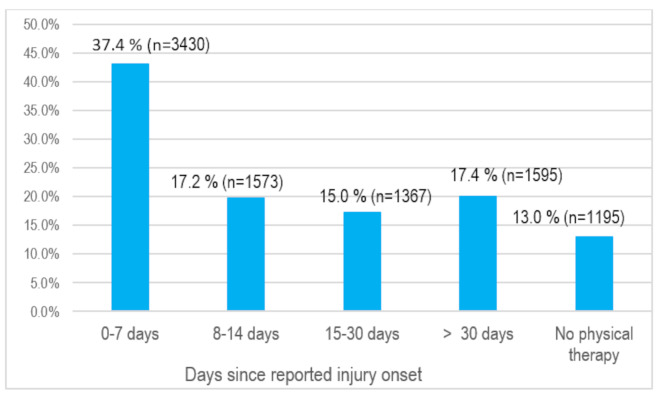
Distribution of PT timing by percentages (*N* = 9160)

Of the included participants, 37.4% (*n* = 3430) of the claimants attended PT within 7 days of reported injury onset. Among the claimants, 17.2% (*n* = 1573) had their first PT visit between 8 and 14 days, 15.0% (*n* = 1367) between 15 and 30 days and 17.4% (*n* = 1595) more than 30 days after injury (Fig. 1).

### Median duration of work disability by PT initiation (unadjusted)

**Fig. 2:**
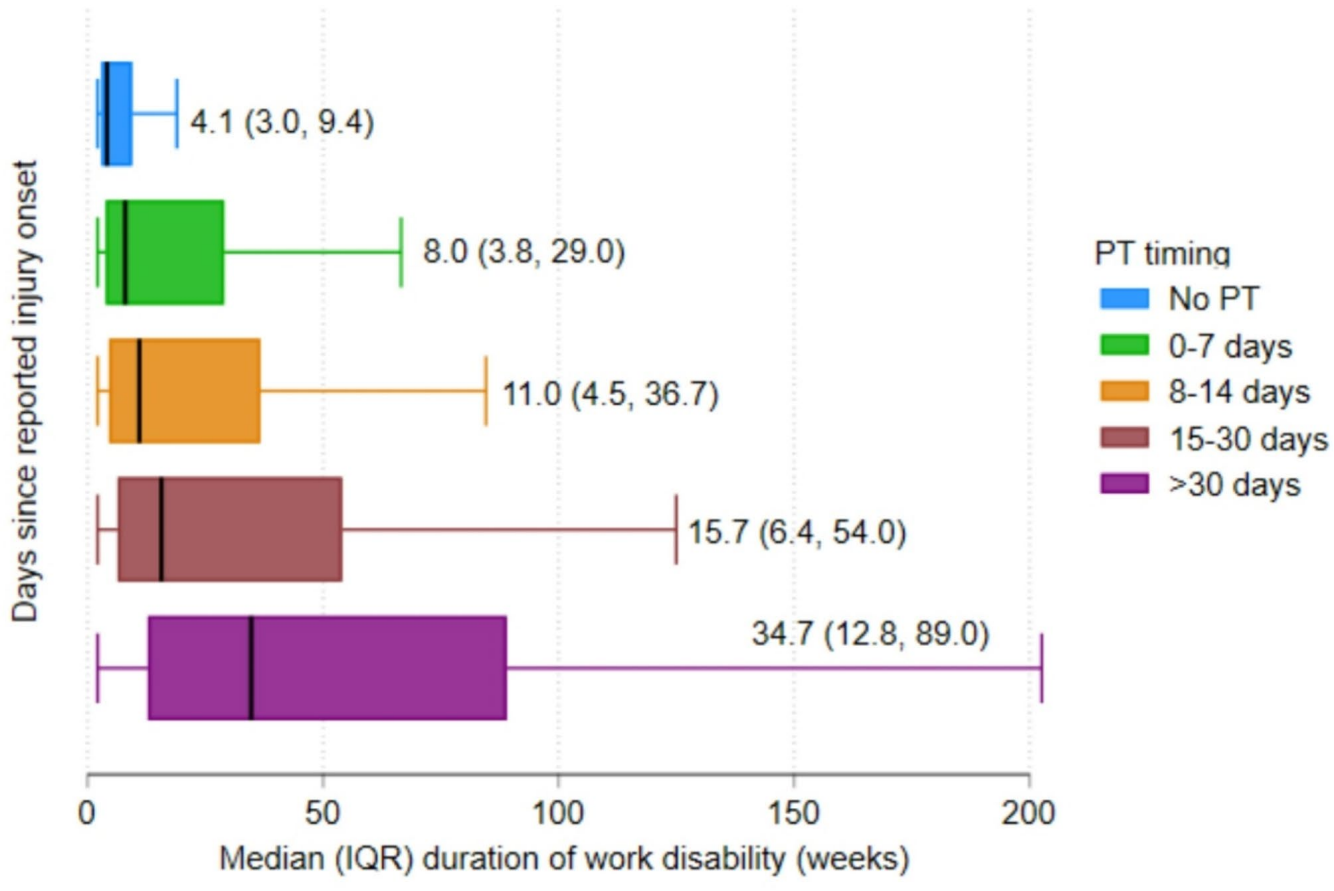
Median (IQR) duration of work disability by the commencement of PT

The overall median duration of work disability was 11.0 weeks (IQR 4.2, 41.4). The median duration of work disability was 8.0 (IQR 3.8,29.0) weeks among individuals who attended PT within 7 days of experiencing back pain. Claimants whose first PT attendance occurred 8–14 days following their LBP exhibited a median work disability duration of 11.0 weeks (IQR 4.5,36.7) (Fig. 2)

### Findings of the adjusted AFT model

**Fig. 3:**
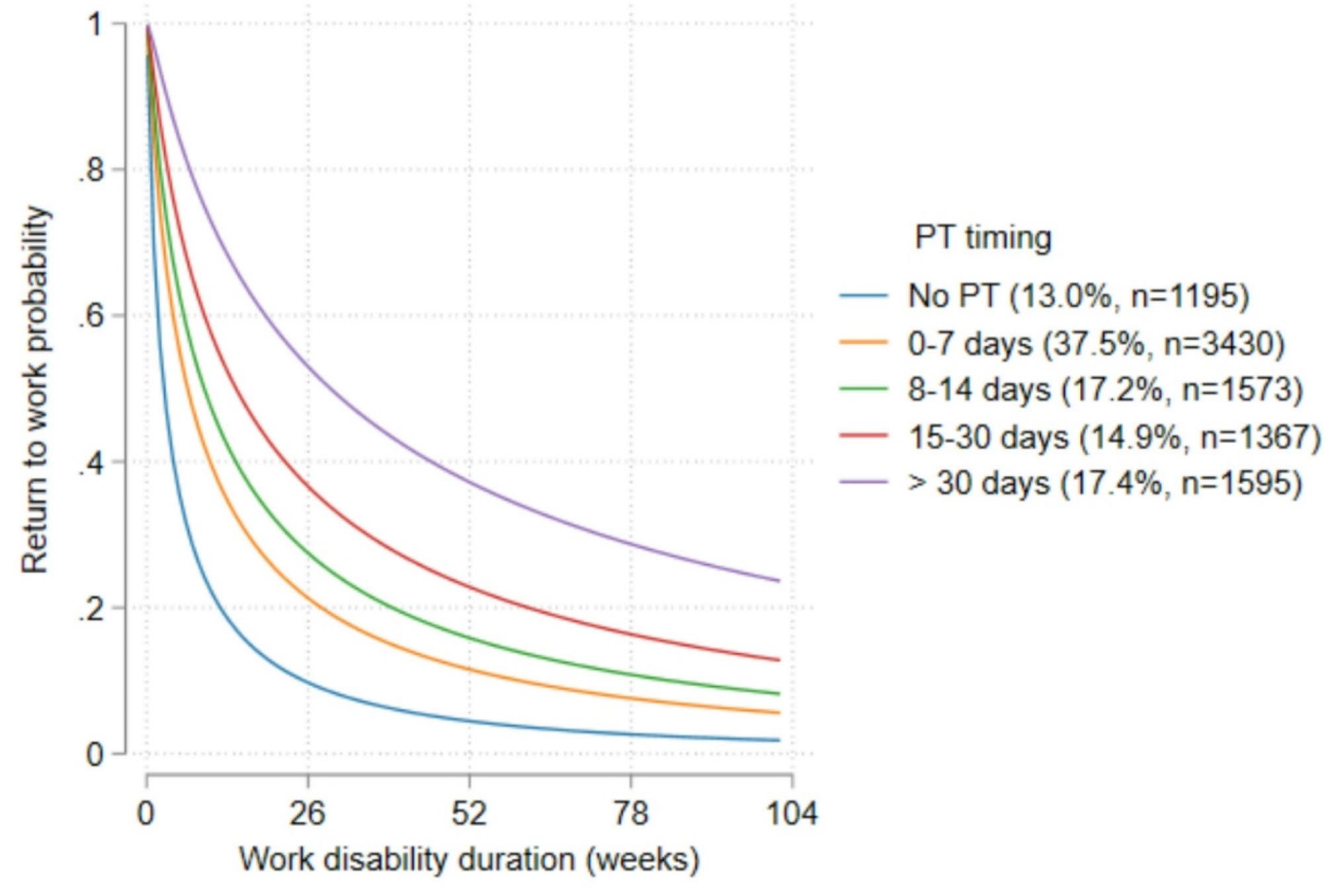
Adjusted survival curve comparing the duration of work disability (in weeks) by PT initiation (in days) after the date of injury

Table 1 shows the findings of the adjusted AFT model for the timing of PT initiation and worker characteristics associated with work disability duration. In this model, PT initiation between 8 and 14 days compared to PT within 7 days post-injury was associated with a significantly longer duration of work disability (TR 1.37; 95% CI (1.23, 1.52)). Similarly, the duration of work-disability was twice as long for a PT commenced between 15 and 30 days compared to a timing of PT within 7 days following the onset of LBP (TR 2.19; 95% CI (1.96, 2.44)). Moreover, the duration of work disability was four times longer in those who attended PT more than 30 days after the date of injury compared to those who attended PT within 7 days of injury (TR 4.51; 95% CI (4.06, 5.02)). Compared to PT timing of 0–7 days, those who did not attend PT had a significantly reduced duration of disability (TR 0.46; 95% CI (0.36, 0.46)) (Fig. 3).

**Table 1:** Sample characteristics, and adjusted AFT model for the timing of PT commencement and worker characteristics associated with work disability duration (*n* = 9160)

Characteristics	Number of claims	Median duration of disability (weeks)	IQR	Adjusted TR (95% CIs)		*P*-value*
				Non-imputed data	Imputed data	
**All**	9160	11.0	4.2, 41.4	**-**	**-**	**-**
**Physical therapy initiation timing**						
0–7 days	3430	8.0	3.8, 29.0	Ref	Ref	Ref
8–14 days	1573	11.0	4.5, 36,7	1.40 (1.25,1.56)	1.37 (1.23,1.52)	< 0.001
15–30 days	1367	15.7	6.4,54.0	2.18 (1.94,2.45)	2.19 (1.96,2.44)	< 0.001
> 30 days	1595	34.7	12.8, 89,0	4.48 (4.01,5.01)	4.51 (4.06,5.02)	< 0.001
No PT care	1195	4.1	3.0, 9.4	0.42 (0.37,0.47)	0.41 (0.36,0.46)	< 0.001
**Age**						
15–25 years	1192	7.8	3.7,24.5	0.63 (0.56,0.72)	0.62 (0.55,0.70)	< 0.001
26–35 years	2238	11.0	4.4,42.1	0.92 (0.83, 1.03)	0.91 (0.83, 1.01)	0.103
36–45 years	2413	11.7	4.5,46.5	Ref	Ref	Ref
46–55 years	2188	11.8	4.4,46.4	0.93 (0.85,1.06)	0.95 (0.85,1.05)	0.323
> 55 years	1129	12.0	4.4,12.0	0.93 (0.82,1.06)	0.93 (0.82,1.05)	0.260
**Gender**						
Female	3027	12.0	4.8, 40.1	1.14 (1.03,1.26)	1.13 (1.02,1.24)	0.011
Male	6133	10.2	4.1, 42.0	Ref	Ref	Ref
**Year of injury**						
2011	1249	11.0	4.2, 43.5	0.96 (0.85,1.09)	0.96 (0.86,1.09)	0.609
2012	2502	11.4	4.4, 43.4	Re	Re	Ref
2013	2543	11.4	4.4, 43.7	1.00 (0.90,1.10)	0.99 (0.90,1.09)	0.902
2014	2069	10.0	4.2, 36.1	0.85 (0.77,0.95)	0.84 (0.76,0.93)	0.001
2015	797	10.1	4.4, 39.2	0.98 (0.85,1.14)	0.97 (0.84,1.12)	0.714
**Jurisdiction**						
South Australia	3353	10.4	4.2,40.5	0.98 (0.90,1.06)	1.08 (0.99,1.17)	0.057
Western Australia	5807	11.2	4.4, 42.0	Ref	Ref	Ref
**Occupation**						
Clerical and Administrative Workers	238	11.7	4.5, 43.5	0.91(0.71,1.17)	0.91 (0.71,1.15)	0.444
Community and Personal Service Workers	1502	10.7	4.4,33.0	0.90 (0.79,1.02)	0.86 (0.76,0.98)	0.026
Labourers	2164	10.4	4.1, 41.4	Ref	Ref	Ref
Machinery Operators and Drivers	2066	11.2	4.2, 44.2	1.00 (0.90,1.12)	0.98 (0.88,1.09)	0.769
Managers	269	11.7	4.2, 44.8	0.96 (0.75,1.21)	0.93 (0.74,1.16)	0.541
Professionals	531	12.5	5.0, 45.0	1.08 (0.90,1.29)	1.03 (0.87,1.23)	0.690
Sales Workers	459	13.0	5.4,45.7	1.15 (0.95,1.40	1.08 (0.90,1.30)	0.360
Technicians and Trades Workers	1932	10.1	4.1, 40.4	0.99 (0.88,1.11)	0.94 (0.84,1.05)	0.322
**Socioeconomic status**						
Most advantaged quintile	1524	10.3	4.4, 34.0	0.93 (0.84,1.03)	0.96 (0.87,1.07)	0.543
Second to fourth quintiles	5421	10.8	4.2,40.8	Ref	Ref	Ref
Most disadvantaged quintile	1270	12.0	4.4,46.5	1.23 (1.09,1.37)	1.16 (1.03,1.30)	0.011
Missing	945	11.5	4.5,49.0	-	-	-
**Remoteness**						
Major Cities of Australia	7053	11.0	4.4, 41.1	Ref	Ref	Ref
Regional Australia	868	10.0	4.1, 42.2	0.84 (0.74,0.95)	0.85 (0.75,0.97)	0.016
Remote Australia	300	8.7	4.2,23.7	0.82 (0.67,1.01)	0.85 (0.70,1.05)	0.142
Missing	939	11.4	4.4,47.8	**-**	**-**	**-**

**Key**: *= p-value indicates the statistical difference between groups, AFT = Adjusted failure time, CIs = confidence intervals, IQR = interquartile range, PT = physical therapy

Regarding demographic and contextual factors, we found a significantly longer work disability for female claimants than for males (TR 1.13; 95% CI (1.02, 1.24)). Our adjusted AFT model also revealed a significantly reduced duration of disability for those aged 15–25 years compared to 36–45 years (TR 0.62; 95% CI (0.55, 0.70)), for injuries occurring in 2014 compared to those in 2012 (TR 0.84; 95% CI (0.76, 0.93)), and for claimants in regional Australia compared to those in major cities (TR 0.85; 95% CI (0.75, 0.97)).

## Discussion

This retrospective cohort analysis investigated the impact of PT initiation timing on the duration of work disability in injured workers presenting with LBP in two Australian states. Median duration of disability was 11.0 weeks, with a median duration of 8.0 weeks in claimants who initiated PT within the first seven days of their injury, compared to a median duration of 34.7 weeks for those who commenced PT more than 30 days post-injury. Claimants who did not use PT had a shorter disability duration, with a median of 4.1 weeks. After accounting for several confounding factors, in those who had PT, attending PT earlier in the claim was associated with a significantly shorter duration of disability after an injury. Moreover, minor variations in the length of disability across other factors have been observed. This study suggests that, among individuals who had PT, early commencement following injury onset may reduce the likelihood of extended disability and accelerate return to work for LBP.

Our study indicated that injured workers with no PT had fewer days off work compared to those who used PT soon after injury. Multiple arguments may support this finding, including individual use of alternative healthcare funding options, injury severity, and access to early return-to-work services. First, some workers may have seen PT outside the workers’ compensation systems, even before filing or having their claims accepted. These individuals may have paid for the services out of pocket or via other insurance and did not claim them through the workers’ compensation scheme, and thus, the data cannot be captured within the workers’ compensation database [42]. It is also possible that other primary care clinicians (e.g. general practitioners (GPs)) may have provided reassurance to injured workers about the favourable prognosis of LBP, including information and education to remain active and continue working, according to best practice recommendations [43–45]. So, the potential use of PT early in the claim process, outside the workers’ compensation schemes or the reassurance offered by GPs, may explain the least disability days observed among those who did not receive PT.

Second, non-PT claims may involve injuries of lesser severity which typically resolve naturally after a few weeks of symptom onset (self-limiting) [4], making treatment unnecessary or minimal for some. Greater injury severity and higher pain intensity are associated with increased healthcare use compared to less severe injuries [46]. Another probable reason could be individual differences in coping mechanisms after an injury. Injured workers who did not use PT may return to work faster because they might exhibit greater self-efficacy — the belief that they can control and self-manage their symptoms without formal treatment [47]. Third, observed differences could be attributed to workplace context, such as the availability of early workplace return-to-work programs [24, 48]. Most workers’ compensation schemes in Australia offer return-to-work services, including modified or alternative jobs [49], which may facilitate an earlier return to work without needing PT. Although the practicability of early return to work services may vary in certain contexts, evidence highlights such initiatives may be effective in reducing the duration of disability [48, 50]. However, our result diverges from the findings of a systematic review and meta-analysis by McDevitt et al. [16]. In that study, a significantly reduced short-term disability (up to six weeks) with small effects was indicated among LBP patients who received PT early in the acute stage compared to non-PT users. Methodological disparities, including study population and outcome measures, may account for the divergent results observed across the studies.

For individuals who attended PT, our findings corroborate multiple studies indicating that early commencement of PT after LBP is associated with a faster return to work. However, differences across various workers’ compensation schemes may limit direct comparison. According to a study by Ehrmann-Feldman et al., LBP cohorts who commenced PT early within 30 days of physician referral showed a significantly increased likelihood of returning to work within 60 days after injury, compared to delayed PT (initiated after 30 days) [51]. Another study by Zigenfus et al. [52] demonstrated that initiating PT on the same day or the day after an injury (Group I) significantly reduced the average time loss compared to delayed interventions (Group II and Group III). Group II commenced their initial PT session between two to seven days, whereas Group III initiated the first session eight days or more after the injury. However, the authors found no significant difference between the later groups, maybe due to other confounders, such as psychosocial factors. Our study demonstrated that among individuals who attended PT, delaying its initiation after an injury was consistently associated with a significant increase in the length of disability across various PT timing.

### Implications

This study highlights the potential value of early PT commencement for injured workers in the Australian workers’ compensation system context. It suggests the need for further research to understand the different recovery trajectories after an injury, including those who returned to work faster without early PT and those who delayed it and sought PT later. Moreover, for injured workers experiencing a delayed PT initiation, increasing awareness of PT consultation for LBP as a primary contact point would likely reduce the delays in returning to work and ease the burden on other healthcare professionals (e.g. GPs) [53]. Previous research highlights that direct access to PT significantly reduces GPs’ workload (e.g. may reduce the need for GPs’ referrals and shorten the timing of PT commencement) [54]. Integrating information and educational resources into workplace programs from the beginning and throughout the claim management process in an occupational context could promote open access to PT and facilitate the timely initiation of treatment [55]. Another potential strategy to promote the prompt uptake of PT among injured workers is to enhance reimbursement incentives for practitioners. For example, WorkCover Queensland has increased clinicians’ incentives by raising the ‘consumer price index (CPI)’ by about 4 per cent, effective July 01, 2024 [56]. This modification may enhance clinician retention and recruitment by boosting their professional motivation and recognising the value of their contribution to patient care, while also improving return-to-work outcomes.

### Limitations and strengths

This study used a large database from two jurisdictions and appears to be the first within the Australian workers’ compensation context to investigate the impact of PT timing on rehabilitation outcomes. To allow for a more comprehensive assessment of the timing of PT care, we also compared the effect of PT at different time points using a rigorous statistical approach. However, some limitations should be noted. Firstly, although some delays in accessing the administrative dataset is relatively common, our data might not precisely represent the current policies in practice in certain states. For example, the South Australian workers’ compensation scheme underwent policy changes in 2015, including eligibility for long-term benefits. Although it is likely for certain states to implement their own workers’ compensation policy reforms, the core feature of work disability insurance, including the ‘caused-based’ approach, has remained consistent across states in Australia and beyond [17, 57]. Future studies should evaluate whether our findings remain reliable in light of these changes in those states. Secondly, while our investigation included cases with a minimum two-week absence from work, symptom severity was not included in the administrative database. Hence, our findings may be influenced by confounding by indications where individuals who receive physiotherapy are likely to have more severe injuries or functional limitations, which may independently contribute to longer work disability durations rather than reflecting a direct effect of physiotherapy itself. In contrast, individuals with less severe injuries or those highly motivated to return to work may recover without physiotherapy or seek alternative treatment options outside the workers’ compensation system. This may, in part, explain the shorter observed disability durations among those who did not receive physiotherapy. Thirdly, this study did not specify the treatment approaches used by physical therapists in their practices. So, this study discussed only within the broader context where PT is used as a profession, rather than a treatment modality. Fourthly, our data predominantly focused on PT funded under workers’ compensation insurance. Injured Australian workers have other potential avenues for the funding of healthcare services for their work-related injuries and conditions, including ‘Medicare’, ‘self-funding’, and ‘private health insurance’ [42]. Although workers’ compensation insurance is typically the primary source of funding during individual claims, it is possible that claimants attended PT that was underwritten by alternative funding sources, for which data is inaccessible. Further limitations of the MJD were presented earlier [27, 32, 58]. Lastly, our analysis was limited to the workers’ compensation status. Consequently, it is unlikely that the findings can be applied to non-compensable injuries or the general population.

## Conclusion

Our study confirmed that initiating PT earlier, rather than later after the onset of compensable LBP was associated with a significantly shorter duration of work disability in those who had PT. Although we cannot establish causality, our findings highlight the potential benefits of initiatives that promote timely PT initiation in reducing the burden of work-related disability.

## Electronic supplementary material

Below is the link to the electronic supplementary material.

Supplementary Material 1

## Data Availability

The datasets analysed during the current study are not publicly available due to confidentiality issues, but the STATA analysis code used to analyse the data is available from the corresponding author upon reasonable request.

## Abbreviations

AFT: Accelerated failure time
CIs: Confidence intervals
GPs: General Practitioners
LBP: Low back pain
MJD: Multijurisdiction database
PT: Physical therapy
TR: Time ratios
