# Supplementary material for: Relationship between the timing of physical therapy commencement and the duration of work disability: a retrospective cohort analysis of work-related low back pain claims
Source: BMC Public Health. 2025 Apr 9;25:1329. doi: 10.1186/s12889-025-22574-x (PMC11983916; doi:10.1186/s12889-025-22574-x)
Supplement: Supplementary file 1 — Supplementary Material 1 [file 12889_2025_22574_MOESM1_ESM.docx]

**Supplementary file 1.** Type of occurrence classification system version 3.1 codes for a low back pain definition

| Nature of Injury | 228 – Trauma to muscles and tendons, not elsewhere classified  229 – Trauma to muscles and tendons, unspecified  239 – Soft tissue injuries due to trauma or unknown mechanisms with insufficient information to code elsewhere  422 – Disc displacement, prolapse, degeneration, or hernia  459 – Back pain, lumbago, and sciatica  533 – Muscle/tendon strain (non-traumatic) |
| --- | --- |
| Location of Injury | 311 – Lower back |
| Mechanism of Injury | Any |
| Agency of Injury | Any |
